# Occupational Exposure to Formaldehyde and Cancer Risk Assessment in an Anatomy Laboratory

**DOI:** 10.3390/ijerph182111198

**Published:** 2021-10-25

**Authors:** Dragan Adamović, Zoran Čepić, Savka Adamović, Milena Stošić, Boris Obrovski, Slobodan Morača, Mirjana Vojinović Miloradov

**Affiliations:** 1 Department of Environmental Engineering and Occupational Safety and Health, Faculty of Technical Sciences, University of Novi Sad, 21000 Novi Sad, Serbia; draganadamovic@uns.ac.rs (D.A.); milenastosic@uns.ac.rs (M.S.); borisobrovski@uns.ac.rs (B.O.); miloradov@uns.ac.rs (M.V.M.); 2 Department of Graphic Engineering and Design, Faculty of Technical Sciences, University of Novi Sad, 21000 Novi Sad, Serbia; adamovicsavka@uns.ac.rs; 3 Department of Industrial Engineering and Engineering Management, Faculty of Technical Sciences, University of Novi Sad, 21000 Novi Sad, Serbia; moraca@uns.ac.rs

**Keywords:** indoor formaldehyde exposure, occupational exposure, anatomical specimens, unhealthy workplace, cancer risk

## Abstract

Dissecting a human cadaver is an irreplaceable practice in general training of medical students. Cadavers in anatomy laboratories are usually preserved in formalin, an embalming fluid whose basic component is formaldehyde (FA). The aim of this study is to assess the cancer risk of employees and students that are exposed to FA based on the results of three monitoring campaigns, as well as to suggest permanent solutions to the problem of FA exposure based on the results obtained. Three sampling campaigns of formaldehyde concentration in indoor environments were conducted at five different locations at the Anatomy Department of the Faculty of Medicine with the purpose of assessing permanent employees’ and medical faculty first year students’ exposure to FA. Indoor air was continuously sampled during 8 h of laboratory work and analyzed in accordance with the NIOSH Method 3500. Exceeding of the 8 h time-weighted average (8 h TWA) values recommended by Occupational Safety and Health Administration (OSHA) of 0.75 ppm was recorded in 37% of the samples during the three-month monitoring campaign. Cancer risk assessment levels for permanent employees were in the range from 6.43 × 10^−3^ to 8.77 × 10^−4^, while the cancer risk assessment levels for students ranged from 8.94 × 10^−7^ to 1.83 × 10^−6^. The results of the research show that cancer risk assessment for employees is several thousand times higher than the limit recommended by the EPA (10^−6^) and point to the importance of reducing exposure to formaldehyde through the reconstruction of the existing ventilation system, continual monitoring, the use of formaldehyde-free products, and plastination of anatomical specimens.

## 1. Introduction

The dissection of human cadavers is an irreplaceable practice in general training of medical students and doctors in specialist training, in conducting research on fundamental anatomical and pathological phenomena, and in the improvement of diagnostic and therapeutic methods. Cadavers in anatomy laboratories are usually preserved in formalin [1], an embalming fluid that contains formaldehyde (FA) as a principal component [2]. Because FA easily polymerizes at high concentrations and after long storage periods, it is often used commercially as a 37–40 % solution of FA (formalin) [3]. Formalin allows long-term tissue stability and it preserves tissue architecture [4]. The term “formalin” describes aqueous solutions that usually contain both FA and an alcohol stabilizer [5,6]. During anatomy dissection courses, FA vapors are emitted from the cadavers, resulting in the exposure of medical students and their teaching instructors to elevated levels of FA and other potentially hazardous components of the preservative solutions. The level of exposure to those agents depends on the duration of time spent in the anatomy laboratory, the working conditions, and the type of embalming performed.

Previous studies actually point to the high formaldehyde concentrations during anatomical courses. Concentration values reach up to 3.1 [7], 3.4, or even 9.16 ppm [8]. Exposure levels in anatomy and pathology laboratories have always been extremely high, often exceeding the 2 ppm short-term exposure limit (STEL), primarily due to the evaporation of formaldehyde from the solution used for the preservation of tissues and specimens [9]. One study found that even when anatomy laboratories were not in use, minimum FA concentrations were still above 0.25 mg∙m^−3^, with a few extreme instances measuring between 13.01 and 20.94 mg∙m^−3^ [10]. In a study conducted in pathological laboratories in Shanghai, the average FA concentration was 1.60 mg∙m^−3^, with the highest value of 5.84 mg∙m^−3^ [11]. FA concentration levels ranged from 0.11 to 1.07 mg∙m^−3^ in the cadaver storage room and 0.06–1.12 mg∙m^−3^ in the gross anatomy laboratory in a Medical College in India in 2012 [12].

Even though FA exposure in anatomy laboratories may occur by direct contact with the eyes or through the skin [13], inhalation is the dominant source of exposure due to the high volatility of FA from the embalmed tissue and its immediate proximity to the breathing zone of the students and instructors [14,15,16,17].

Most of the inhaled FA is deposited and absorbed in the upper respiratory tract, which the substance comes into contact with first [18]. Once absorbed, FA is very quickly broken down [19]. Almost every tissue in the human body has the ability to break down FA to less toxic products [20]. Enzymes decompose FA to formic acid, which is excreted via urine. Formic acid can be further broken down into carbon dioxide and breathed out of the body.

Exposure to FA solutions can cause irritation of the skin and allergic contact dermatitis [21,22]. FA leads to eye irritation, a dry or sore throat, mucous membranes, a tingling sensation of the nose, menstrual disorders, and pregnancy problems [23,24,25]. It is highly irritating to the upper airways, and FA-induced asthma may result from a strong allergic response [26].

In the case of prolonged exposures, FA has been related to an increased risk of squamous-cell carcinomas of the nasal cavities in animals and nasopharyngeal cancers in humans [26]. Epidemiological studies of FA-induced cancer have been extensively conducted, but the mechanism of carcinogenesis has not been fully clarified. Zhang et al. [27] proposed three potential mechanisms of FA-induced leukemia: direct damage to bone marrow stem cells through the blood, damage to hematopoietic stem cells circulating in the blood, and damage to primitive pluripotent stem cells present in the nasal or oral passages [28]. Since FA and its metabolites naturally exist in all cells, FA toxicity likely occurs as a result of exposure to high concentrations that exceed normal metabolic capacities [29,30,31].

Although FA is a natural metabolic product of the human body, high-dose exposure increases the risk of acute poisoning, while prolonged exposure can lead to chronic toxicity and even cancer [32,33]. Based on comprehensive research and large-scale international human studies, the International Agency for Research on Cancer (IARC) has classified FA as a Group 1 human carcinogen that causes nasopharyngeal cancer and probably leukemia [32,34].

The purpose of this research was to conduct an air sampling study in order to determine if the concentrations of FA present in the air during the common practice in different rooms of the Anatomy Department have exceeded the permissible exposure limits recommended by international expert organizations including the U.S. OSHA (Occupational Safety and Health Administration) established concentration levels. Other goals of this research were to provide relevant data on FA concentration levels during the standard teaching process and to assess the cancer risk of students, teaching, and non-teaching staff residing on the premises of the Department based on the FA concentration levels detected and the duration of exposure of the corresponding categories.

## 2. Materials and Methods

### 2.1. Description of Sampling Locations

The Department of Anatomy is located on the ground floor of the Medical Faculty, Novi Sad, Serbia. The classes and practical exams in anatomy are held in the classrooms of the department, which are located near the department’s entrance. The teaching cabinets, laboratories, and facilities for housing, keeping, and preservation of anatomical specimens are located in the other part of the department, opposite the main entrance.

The practical classes are conducted in two classrooms with 10 tables for dissection of anatomical specimens in each room. The department employs 13 professors, 4 lab technicians, 2 technical assistants, and 4 members of the cleaning staff. Anatomical specimens are stored in a separate room. While not in use, anatomical specimens are kept in a solution prepared by mixing 1l of formalin, 500 g of glycerine, 500 mL of phenol, and 8 l of water. Three days prior to each block of exercises, the specimens are taken out of the formalin and rinsed in the preparation room. A technical assistant takes specimens out of the formalin, and at the given moment he is wearing a gas mask in order to prevent the exposure to extremely high concentration levels of FA. For the duration of the training, students work with dry and wet preparations using only a surgical mask and latex gloves as a means of personal protection.

The dimensions of both classrooms the dissection of anatomical specimens takes place in are 10.3 m × 6.7 m (69 m^2^) with a height of 3.5 m and a volume of 241.5 m^3^. Both are equipped with 10 dissection tables. The air supply is 1700 m^3^/h, and the air exhaust accounts for 1870 m^3^/h. This adds up to 7.7 air changes per hour. Fresh air is delivered by ceiling slot diffusers, and the exhaust air is extracted by ventilation grilles on approximately the same level. The air is not recirculated but exhausted outside, and there is no air damper. There is no ventilation in the storage, preparation, and break rooms and the ventilation in those rooms is done solely naturally.

Quarterly monitoring of FA’s concentration levels at the department’s working premises was conducted within three campaigns. This sampling period covered training exercises with dry (bones) and wet anatomical specimens (specimens stored in formalin) and pre-examination exercises when students worked with the largest number of various anatomical specimens throughout the school year. Pre-examination exercises make a final preparation, that is a final revision of their knowledge before the final exam. In that period, students have at their disposal the highest number of anatomical specimens.

The measurements of concentration levels of FA were conducted at five locations inside the department. Air sampling was conducted in two classrooms, storage room, preparation room, and break room in which the employees, professors, and support technical staff reside during their breaks in the process of conducting classes. For the duration of the campaign, 20 air samples were analyzed at each sampling location, with a total of 100 samples per campaign.

In order to reduce the concentration levels of FA in the department, there is a ventilation system and the windows in the lab are also opened as a method of ventilation. In winter months and low external temperatures, the windows are most commonly shut. Because of this, only exercises with dry anatomical specimens are conducted in this period of the year in order to lower the effect of FA as much as possible. When the external temperatures get higher, exercises with wet anatomical specimens are conducted. In this period of the year, the windows are customarily open.

The temperature in the rooms during the three sampling campaigns ranged from 17 to 22 °C, whereas the relative air humidity ranged from 35% to 48%. In the conditions of higher outside temperatures, during the third campaign performed in May, the room temperature rose to 26 °C and relative air humidity reached 70%.

The first sampling campaign was conducted during February 2018, i.e., in the winter period. The outdoor temperatures during the implementation of the sampling campaign ranged from −2 to 8 °C, while the internal temperatures were in the range of 17–22 °C. During the implementation of the first campaign, training exercises with dry bones of the head were conducted. There were 10 dry anatomical specimens in each classroom.

The second sampling campaign was conducted in March 2018. Outside temperatures during the implementation of the second sampling campaign were in the range of 17–21 °C, which made more efficient airing possible. During the implementation of the second campaign of measurements, training exercises with 20 wet anatomical specimens, which were taken out of the formalin and washed three days before the first class of training, were carried out. Namely, the second campaign was conducted in the early spring period in the conditions of higher outside temperatures compared to the previous campaign. For this reason, the windows towards the courtyard were opened more frequently.

The third campaign was organized in May 2018 at the same time as the extraction of the largest number of anatomical specimens from formalin, within the pre-examination practical training. During the third sampling campaign, the outdoor temperatures were in the range of 26–30 °C, which allowed the windows to always be open during the entire campaign. Students worked with 35 anatomical specimens during the training (10 dry and 25 wet specimens).

In order to determine the possibility of outdoor FA infiltration, control measurements were conducted in the yard and in front of the entrance to the department and the presence of FA was not detected. Therefore, this study solely addressed the five indoor rooms.

### 2.2. Measurements of Formaldehyde Concentration Levels

Indoor air was continuously sampled during 8 h of laboratory work by using the air sampler PRO EKOS 401-x. The device was mounted at a height equivalent to the breathing zone, approximately 1.50 m above the floor. The air was infiltrated through the Drechsel bottles with diffuser frit containing absorption solution for FA. The air flow was set at 0.5 L/min. The device PRO EKOS 401-x was periodically checked regarding the flow rate in the Laboratory for Fluid Mechanics, Faculty of Technical Sciences, Novi Sad. For the purposes of checking the flow rate, the rotameter model 6A0101BV-AB produced by Dakota Instruments with a measuring range from 0.1 to 1 L/min and accuracy of +/−5% was used. The float of the rotameter is made from stainless steel and the maximum pressure and temperature of the rotameter are 700 kPa and 65 °C, respectively.

In the presence of concentrated sulfuric acid, chromotropic acid reacts with FA to give a red-violet hydroxydiphenylmethane derivative. The resulting chromophore can be analyzed by UV/VIS spectroscopy by measuring the absorbance at 580 nm. Upon the sampling completion, it was necessary to immediately determine the FA concentration, as the intensity of the purple color of the absorption solution remains stable only for a few hours. For that reason, the bottles were wrapped in aluminum foil, refrigerated, and then transported to the laboratory, where spectroscopy analyses were performed immediately to minimize the risk of interference.

The samples were analyzed in an accredited laboratory of the Department of Environmental Engineering and Occupational Safety and Health, Faculty of Technical Sciences in Novi Sad by using the UV/VIS spectrophotometer DR 5000 (HACH LANGE, Germany) with proper QA/QC in accordance with the NIOSH Method 3500 [35,36,37], also known as the chromotropic acid method. Using this method, 300 area samples were collected in the workplace and analyzed.

### 2.3. Chemicals, Reagents, and Standards

During the experimental determination of the concentration levels of FA in different premises of the Anatomy Department, the following chemicals, reagents, and standards were used:

- A solution of chromotropic acid. It was prepared by dissolving 0.1 g of chromotropic acid (4,5-dihydroxy-2,7-naphthalenedisulfonic acid disodium salt) in water and diluting to 10.0 mL. The solution was stored in a dark bottle and prepared once a week.
- Concentrated sulfuric acid, p.a.
- Standard solution of FA (1 mg/mL). It was prepared by dissolving 2.7 mL of a 37% FA solution in 1 l of distilled water.
- Working solution of FA (10 µg/mL). It was prepared by dissolving 1 mL of the standard solution in 100 mL of distilled water. This solution is very unstable and needed to be prepared daily.
- Absorption solution. It was prepared by adding 9.5 mL of concentrated sulfuric acid and 0.5 mL of 1% chromotropic acid into the Drechsel bottle.

### 2.4. Standardization of Formaldehyde Solution

Standardization of the FA solution was carried out by diluting 5 mL of the concentrated solution (35–40%) of FA to 250 mL with redistilled water. Then, 5 mL of this solution were taken with the pipette and then added with 40 mL of 0.1 N iodine solution. Then, 6N NaOH was added dropwise until the solution became pale yellow. The prepared solution rested for 10 min. It was then acidified with 20% HCl, and excess iodine was titrated with 0.1 N sodium thiosulphate. The same procedure was done with a blank solution, only in the blank solution, 5 mL of redistilled water were added instead of the FA solution. It was necessary to record the volume of consumed sodium thiosulphate in the sample and in the blank, and then detract the ml of the spent sodium thiosulphate sample from the blank sample. One milliliter of 0.1N J_2_ corresponded to 1.5 mg of FA because the reaction took place according to the equation:

HCHO + H_2_O + I_2_ → 2HI + 2HCHO (1)

The concentration of FA in the original solution was calculated by the equation:

HCHO mg/mL = 10 × 1.5 × mL 0.1N I_2_ (2)

The working solution of FA was prepared by diluting the standard solution of FA with distilled water so that 1 mL of the working solution contained 10 μg of FA.

### 2.5. Initial Calibration

The calibration standard solutions were prepared by pipetting 10, 20, 30, 50, and 100 μL of the working solution of FA, and poured into 10 mL of the absorption solution.

The concentration of FA in the prepared standards was: 1, 2, 3, 5, and 10 μg in 10 mL of absorbent solution.

The concentrations of FA were determined from the calibration curve by using the standard FA solution. The FA concentrations in air were calculated based on the determined concentrations of FA and the amount of air transmitted through the absorbing solution.

### 2.6. Calculation

(3) $$\frac{\mu g}{m^{3}}HCHO=\frac{A\times F}{V}$$

where:

*A*—absorbance of the sample.
*F*—slope of the calibration curve.
*V*—volume of air infiltrated through the Drechsel bottles in m^3^, calculated at a temperature of 25 °C and a pressure of 1013 mbar.

Conversion factor at 1013 mbar and 25 °C, 1 ppm = 1.228 mg m^−3^.

### 2.7. Quality Control

A blank test was performed daily before the analysis. A blank test was made by adding 0.5 mL of 1% chromotropic acid to 9.5 mL of concentrated sulfuric acid.

After the completion of the sampling, the absorbance of the sample was compared to the blank test on a spectrophotometer at a wavelength of 580 nm in a 2 cm cuvette.

Verification calibration was performed at least once a month and it was determined whether the results obtained deviated from the specified values. The results of the verification calibration must be within the range of +/−10% of the initial calibration value.

### 2.8. Statistical Analysis

The monitoring results were subjected to statistical analysis. The analysis of results was performed with IBM SPSS v21 (IBM SPSS Statistics for Windows, Version 21.0. Armonk, NY, USA: IBM Corp.), separately for each location. Conformity observed distributions with the theoretical normal distribution were analyzed by using the values of skewness and kurtosis, and the homogeneity of variance for tested results of measurements was analyzed by using Levene’s test. The results were analyzed with analysis of variance (ANOVA) and post-hoc Tukey’s test; *p* = 0.05 was adopted to determine the significance of differences between monitoring results for the different locations.

### 2.9. Cancer Risk Evaluation

Considering the high concentration levels of FA, which is continuously present in different rooms of the department, risk assessment regarding the teachers, technical assistants, cleaning staff, and students was conducted.

As part of the health risk assessment, as a consequence of exposure to FA, evaluation of cancer risk was conducted on the employees of the department, as well as the students who attend anatomy classes as an obligatory course at the first year of the Faculty of Medicine.

The probability of an individual developing cancer over a lifetime (CR) was estimated by multiplying the cancer slope factor (SF) by the chronic daily intake (CDI) according to the Integrated Risk Information System (IRIS) [38]. The cancer slope factor is a plausible upper-bound estimate of the probability that an individual will develop cancer as a result of lifelong exposure to a particular level of a potential carcinogen [39].

The slope factor (SF) turns the expected value of a daily intake of a substance throughout one’s lifetime directly into the risk of developing cancer. If we assume that the slope factor is constant, the risk is directly related to the intake:

(4) CR=CDI×SF

According to the IRIS system, the slope factor in this study of FA is 0.0455 (mg/kg/day)^−1^ [40,41,42].

For the calculation of the CDI, certain values were assumed according to USEPA [43]:

(5) $$\mathrm{CDI}=\frac{\left(C\times \mathrm{IR}\times \mathrm{ED}\times \mathrm{EF}\times L\right)}{\left(\mathrm{BW}\times \mathrm{ATL}\times \mathrm{NY}\right)}$$

The values used for the calculation of CDI of employees and students are shown in Table 1.

IR of 1.02 m^3^∙h^−1^ (average inhalation) was used, in accordance with the Exposure handbook factors [43,44].

During the calculations of cancer risk, the fact that the first-year students are exposed to FA 4 h a week over 30 teaching weeks, and the fact that the student attendance at the premises of the department is limited to classrooms 1 and 2 were taken into consideration. The levels of cancer risk of employees and students were calculated separately during all three measuring campaigns for every individual room.

## 3. Results

The results of the measurement of FA concentrations during all three sampling campaigns are shown in Table 2.

### 3.1. The First Sampling Campaign: Dry Anatomical Specimens

**Figure 1:**
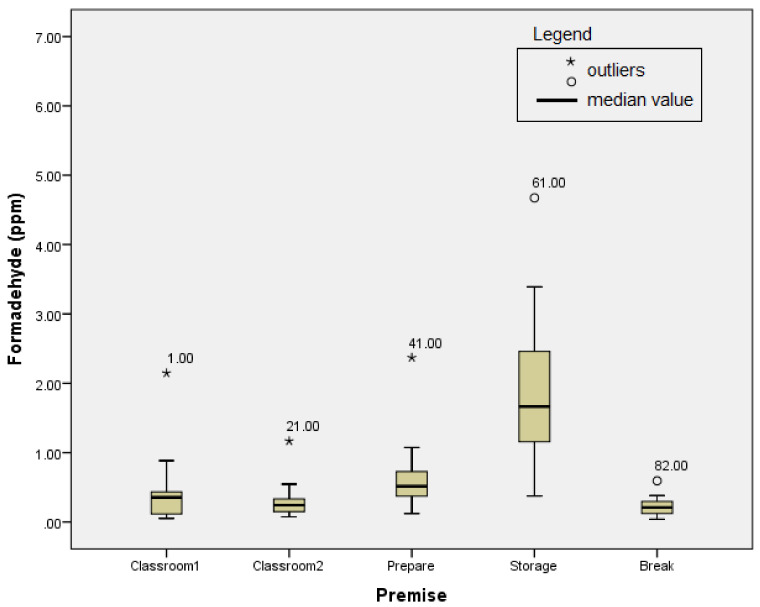
The results of the first sampling campaign.

The results of the measurements are shown in Figure 1. In working with dry anatomical specimens, the lowest mean 8 h-TWA (time-weighted average) FA values in all of three campaigns were detected. Nonetheless, the recommended limit for the eight-hour workday by OSHA of 0.75 ppm was exceeded at all sampling points except the break room, where the lowest campaign median value of 0.04 ppm was detected.

In order to make a more objective assessment of the obtained results of the measurement of FA concentration levels, all outliers were identified and eliminated from further statistical analyses. After removing the outliers, all variables satisfy the assumption of the distribution normality concerning the skewness and kurtosis values. The highest average value of the FA TWA concentration during the first campaign was observed in the storage room, which was identified as the dominant source of emission during the first sampling campaign.

Besides the high mean value, the highest eight-hour value of 4.67 ppm of the entire first campaign was also detected in the storage room (Figure 1).

### 3.2. The Second Sampling Campaign: Wet Anatomical Specimens

**Figure 2:**
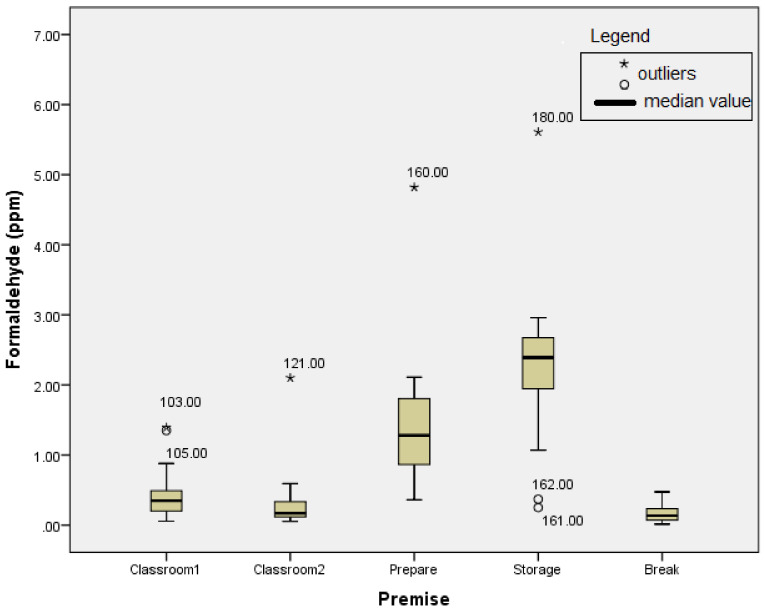
The results of the second sampling campaign.

The results of the second campaign are shown in Figure 2.

During the second campaign, higher average concentrations of FA were observed at all sampling points when compared to the previous campaign, except for the average concentration in the break room, probably as a result of more efficient airing (Table 2). The mean FA concentration values detected surpassed the mean values prescribed by OSHA in the storage and preparation room. The main reason for this occurrence is the use of wet anatomical specimens. The highest TWA concentration of FA during the second sampling campaign of 5.61 ppm was recorded in the storage room, like in the previous campaign. The lowest value of the FA TWA concentration during the whole campaign of 0.01 ppm was detected in the break room.

### 3.3. The Third Sampling Campaign: Dry and Wet Anatomical Specimens

**Figure 3:**
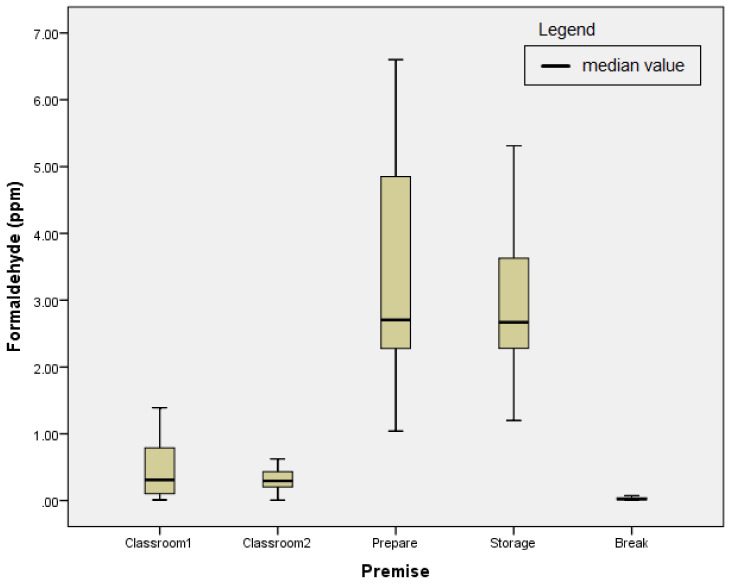
The results of the third sampling campaign.

The results of the measurements carried out within the third campaign are shown in Figure 3.

During the third FA sampling campaign, outliers were not identified in the measurement results. Assumptions about the normal distribution of the measured values of concentrations were satisfied at all sampling points.

The highest TWA concentrations of FA during this campaign were recorded in the preparation and storage rooms as 6.60 ppm and 5.31 ppm, respectively. The preparation room was identified as the most powerful source of emission during the third campaign due to intense preparations of a large number of anatomical specimens, which also makes up for the difference between the concentration levels detected in previous sampling campaigns. Higher average concentrations of FA were also recorded in the classrooms, in comparison with the previous measurement campaigns, as a result of the presence of a large number of wet anatomical specimens. The lowest average concentration of FA (0.01 ppm) during the third measuring campaign was detected in the break room.

## 4. Discussion

FA irritates human tissues and organs when it comes into direct contact with them. The most common symptoms include irritations of the eyes, nose, and throat, along with increased tearing, which occur at air concentrations of about 0.1–1 ppm. FA levels between 0.1 and 0.5 ppm (about 0.12–0.6 mg∙m^−3^) are detectable by human senses, the ones between 0.5 and 1.0 ppm (0.6–1.2 mg∙m^−3^) can cause eye irritation, and those above 1.0 ppm (1.23 mg∙m^−3^) can irritate the nose and throat [45]. The National Institute for Occupational Safety and Health (NIOSH) states that FA is promptly dangerous to life and health at 20 ppm [20].

The maximum allowed concentration (MAC) of FA in Serbia is 0.1 mg∙m^−3^. Serbia’s Air Quality Standard [46] uses the World Health Organization (WHO) recommended threshold for indoor FA concentration [47]. Other countries prefer to use time-weighted averages (TWAs) and/or short-term exposure limits (STELs) rather than MACs. For example, the US OSHA has established limits for the amount of FA that workers can be exposed to at their place of work. At present, the limit is at 0.75 ppm on average over an 8-hour workday (0.92 mg∙m^−3^, 8 h TWA). The highest concentration level that a worker can be exposed to is 2 ppm (2.46 mg∙m^−3^), and that can only occur over 15 min (STEL) [48,49]. While the United Kingdom stands at 2 ppm for both TWA and STEL [50], the American Conference of Government Industrial Hygienists (ACGIH) has set a threshold limit value ceiling (TLV-C) of 0.3 ppm [51].

During the implementation of the three-month monitoring campaign in the various premises of the department, from the 300 samples analyzed, an 8 h-TWA exceeding the value of 0.75 ppm was recorded in 110 samples, representing 37% of the total number of samples. Exceedances of the limit value are most common in the storage and preparation rooms, where, in 97% and 67% of the samples, a concentration higher than OSHA 8 h TWA values was detected. In Classroom 1, the exceedance of the TWA values was less frequent (18%), while the exceedance of the limit value in Classroom 2 was detected in only 3% of the analyzed samples. In the break room, TWA overruns were not recorded over all three monitoring campaigns. When it comes to exceeding the limit value in different campaigns, the measurement results indicate that the overrun of TWA values was recorded in 26% of samples during the first campaign, 38% during the second, and 46% of the samples during the third measurement campaign.

### 4.1. The Comparison of Results Obtained from Three Sampling Campaigns

After carrying out the descriptive statistical analysis, the removal of outliers in order to provide a normal distribution of the data and a more objective assessment of the general conditions at the department, a single-factor analysis of variance was conducted in order to determine the statistically significant difference in the mean values of the concentration levels of FA during the various campaigns.

**Figure 4:**
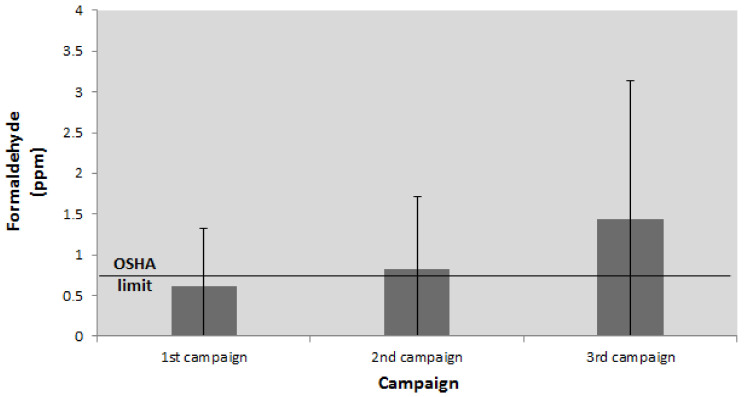
The average FA concentrations during the sampling campaigns.

Using a single-factor analysis of variance, a statistically significant difference in the concentration levels of FA during the three sampling campaigns was detected at level *p* < 0.05. In addition to the detected statistical significance, the size of the actual difference according to Cohen’s criteria [52] indicates the median impact of the various sampling campaigns. The size of this difference is expressed using the indicator η^2^ = 0.08. Subsequent comparisons with the Tukey’s HSD test indicate that there is no statistically significant difference between FA concentrations in the first (M = 0.61 ppm, SD = 0.71) and the second (M = 0.83 ppm, SD = 0.89) sampling campaigns. A statistically significant difference was identified between the FA concentration levels in the second and third campaigns (M = 1.43 ppm, SD = 1.71) and between the results of the first and the third campaigns. This indicates that an increase in the number of anatomical specimens resulted in a statistically significant increase in the average FA concentration at the level of the entire department. The average FA concentration values at the department were above the recommended OSHA value during the second and the third campaigns, while the average FA concentration level during the first campaign was below the recommended value (Figure 4).

**Figure 5:**
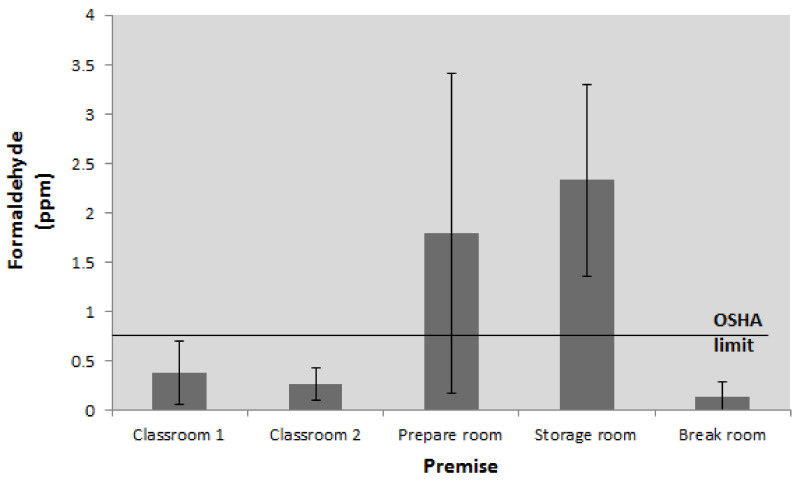
The average FA concentrations in different premises during the sampling campaigns.

In order to identify the workplace most exposed to the influence of FA at the department, a single-factor analysis of variance was carried out to determine the existence of statistically significant differences in FA concentrations in different premises. A statistically significant difference at *p* < 0.05 was found in five different rooms. In addition to the statistical significance, a large effect size was determined according to Cohen’s criteria η^2^ = 0.53. Subsequent comparisons with the Tukey’s HSD test identified the storage room (M = 2.33 ppm, SD = 0.97) as the dominant FA source at the department by comparing the results of all three campaigns, with a statistically significant difference concerning all premises at the department. The concentration of FA in the preparation room (M = 1.79 ppm, SD = 1.62) was also statistically significantly higher than the concentrations in other premises at the department: Classroom 1 (M = 0.37 ppm, SD = 0.32), Classroom 2 (M = 0.26 ppm, SD = 0.16), and break room (M = 0.14 ppm, SD = 0.14) among which statistically significant differences in the concentration levels of FA were not detected. During the third campaign, the preparation room was identified as the most reliable source of FA, due to the extraction of a large number of anatomical specimens from formalin during the preparation of pre-examination training exercises. Despite this fact, the storage room is considered as the most potent source of FA emissions in the department, due to the constant FA emissions throughout the year, and not only during specific periods (Figure 5).

All of the above determines the position of a technical assistant as the most vulnerable when it comes to FA exposure. Due to the type of work, technical assistants spend most of their working hours in storage and preparation rooms where the concentrations of FA outweigh the values recommended by international professional organizations. The cleaning staff represent the next level of exposure to FA. During their working day, they access all premises so that the average concentrations at the department during each campaign, without outliers, were used to evaluate their exposure to FA. The teachers, laboratory technicians, and students who stay and work in the department are mainly limited to classrooms where the lowest FA concentrations were detected during all three sampling campaigns, and were thus exposed to the lowest concentration levels of FA.

### 4.2. The Employees’ and Students’ Cancer Risk

Based on the data of FA concentration levels measured during three measuring campaigns, the cancer risk of the students’ exposure is significantly lower than that of the employees, considering the considerably shorter period of their exposure to FA. The results of the calculations of the cancer risk of employees and students are shown in Table 3.

Considering the fact that the average concentration levels of FA rose during the campaigns, a corresponding increase in cancer risk levels in all rooms was recorded.

The calculations indicate an extremely high level of cancer risk of the employees at the department of anatomy. The values obtained in all rooms are significantly higher than those recommended by international expert organizations. The US Environmental Protection Agency (US EPA) prescribes an acceptable risk level value of the order 10^−6^ (1 in 1,000,000) (KBCHSS, 2001), while NIOSH prescribes a significantly higher level of the order 10^−3^ (1 in 1000) [53]. Generally, US EPA uses the 1 in 10,000 to 1 in 1,000,000 risk range as a target range within which the agency strives to manage risk. The EPA uses the 1 in 10,000 risk level as an appropriate cut-off level for decisions on whether risk management action is required at a site [54]. The results of the calculations are shown in Table 3.

According to the results of FA monitoring during the three sampling campaigns, it can be noticed that the technicians are exposed to the highest risk levels in the storage and preparation rooms (1.01 × 10^−3^ to 6.43 × 10^−3^), especially during the process of extraction of the anatomical preparations from FA, when the cancer risk levels were several thousand times higher than those recommended by US EPA (10^−6^).

The members of the cleaning staff, whose stay and work operations are not predominantly related to the teaching process and a particular location within the department, were exposed to the next, somewhat lower, level of cancer risk. For the exposure assessment of the cancer risk level of members of the cleaning staff, for this reason, the average values of the concentration of FA at the department during the appropriate campaign were used. The estimated levels of cancer risk for the cleaning staff during the three measurement campaigns were in the range from 1.14 × 10^−3^ to 2.67 × 10^−3^.

The cancer risk levels of the teaching staff (professors and laboratory technicians) that conduct the practical training of students are somewhat lower than that of the other employees at the department but mostly uniform during the usual teaching process in the first and second sampling campaigns, as indicated by the values of cancer risk levels obtained in Classrooms 1 and 2 (4.29 × 10^−4^ to 5.78 × 10^−4^). A somewhat higher cancer risk level of the teaching staff was recorded during the third campaign (5.60 × 10^−4^ to 8.77 × 10^−4^) when a more significant number of anatomical specimens were present in the classrooms due to the usual teaching process.

The levels of cancer risk of students were calculated on a yearly scale, whereby students’ attendance that is limited to Classrooms 1 and 2 was taken into consideration. The results are shown in Table 3. When it comes to students, these risk levels are in accordance with those recommended by international organizations (from 8.94 × 10^−7^ to 1.83 × 10^−6^).

### 4.3. Possible Solutions for the Lowering of FA Concentration Levels

Despite the well-known and proven toxicity and carcinogenicity of FA, anatomists and others in Serbia and many other countries, especially developing countries, show little enthusiasm for the reduction of FA in the process of preparation and preservation of anatomical specimens and the identification of equally effective replacement chemicals. The best method for preventing adverse health outcomes would be to provide a working environment free from the hazards. While it is not usually possible to completely eliminate the risk caused by FA, its reduction to the lowest level should be established as an ultimate target. To reach that goal, the collective prevention measures based on the systemic, technical, organizational, and personal measures must be used [55].

Concentration levels of FA should be measured continually, especially during the process of dissection in the anatomy laboratory, to ensure that the allowed concentrations are not exceeded.

Furthermore, the local exhaust ventilation system should be improved. Since FA is heavier than air, vents should be installed below the breathing zone of employees. There is no sense in designing vents in the upper part of the premise and dragging vapors that are heavier than air through the breathing zone of exposed workers and making the existing problem even more difficult. The current state of affairs at the department is such that the ventilation grilles are located in the upper part of the room above the breathing zone and that the possibility of moving the ventilation grilles closer to the floor would require a complete reconstruction of the ventilation system.

Personal protective equipment, such as safety glass and gloves, should be available and used to prevent direct skin or eye contact. Employees should wear nitrile gloves rather than latex because they are more resistant to FA.

It is also recommended that the all-face mask type 3M should be in use, as a last resource, to protect the respiratory system and the eyes of the employees since the ambient air in all areas of the department is contaminated by FA.

The management of the anatomy laboratory should consider replacing high-concentration formalin products with low-concentration or less hazardous or formaldehyde-free products and body preservation fluids that are effective in preventing decomposition of cadavers, maintaining a desired life-like appearance of the body, which are non-hazardous for dissection and environmentally safe [56,57].

One of the possibilities of lowering the concentration levels of dangerous chemicals in air in the anatomy laboratory, along with increasing the quality of teaching, is given by the plastination process of anatomical preparations, like artificial manikins. Plastination offers one of a kind, real human specimens preserved through plastination for medical teaching, anatomy labs, and instruction. All plastinates are odorless and non-toxic, even if handled without gloves. They are durable, flexible, authentic, and do not shrink [58]. The water and fat are substituted by certain plastic biomaterials, yielding specimens that can be touched, do not smell or decompose, and even retain most properties of the original sample [59]. These specimens allow studies and research of the human body without the usual disadvantages of real human bodies like moldering, smell, and the health hazards of FA and other hazardous, emerging, toxic, and carcinogenic substances.

### 4.4. Study Limitations

The experimental research was conducted in real, not controlled, experimental conditions. For the given reason, some parameters were very hard to anticipate, and even harder to control. One of the mentioned parameters was natural ventilation, which is used as a way to ventilate the premises, especially in the conditions of higher outside temperatures, which greatly affects the fluctuations in FA concentration levels during measurement campaigns. CR assessment was conducted based on the time the employees spent in the premises according to the regular working procedure, which does not exclude the possibility of changes in the daily working routine and therefore the discrepancies in real as opposed to estimated values of CR. Additionally, the CR assessment focused on inhalation as the dominant way of exposure to FA. The authors were not in a position to monitor the effects of FA via skin and eyes, which also affects the estimation results obtained to a certain degree. A weakness of the sampling method applied can be observed in the position of the sampler. Given the fact that FA is heavier than air and that it falls to the bottom of the room, the employees inhaling air at 1.50 m and slightly higher are likely to be exposed to somewhat lower concentration levels compared to the measured values. The authors also do not exclude the possibility of photo degradation of FA in the sample from the moment the sampling ended to the moment the laboratory analyses were completed.

The literature limitations are also the vague mechanisms of cancerous disease formation induced by exposure to FA, as well as the lack of long-term clinical data about the prevalence of cancers attributable to FA exposure in anatomy laboratories.

## 5. Conclusions

This study points to the presence of formaldehyde with concentration levels significantly higher than those prescribed by the Serbian legislature and recommended by international expert organizations. This research highlights the irritating action of FA on medical students and the chronic toxic effects on staff members.

Since numerous laboratories worldwide are facing a similar problem, this study emphasizes the importance of reducing exposure to formaldehyde through reconstruction of the existing ventilation systems, continual monitoring, the use of formaldehyde-free products, and plastination of anatomical specimens. Due to the toxic effects of formaldehyde, finding an environmentally friendly and cost-effective alternative is crucial to the health of employees.

## Figures and Tables

**Table 1:** The data for the calculation of cancer risk of employees and students.

Parameter	Description	Value Employees (Students)	Unit
C	Contaminant concentration		mg∙m^−3^
IR	Inhalation rate, adult	1.02	m^3^∙h^−1^
ED	Exposure duration	40 (4)	h∙week^−1^
EF	Exposure frequency	36 (30)	week∙year^−1^
L	Length of exposure	40 (1)	years
BW	Body weight	70/60	kg
ATL	Average time of life, man/woman	69/72	years
NY	Number of days per year	365	days∙year^−1^

**Table 2:** FA concentrations during sampling campaigns.

1st Sampling Campaign (February)							
Premise	Mean	SE	SD	Min	Max	Skewness	Kurtosis
Classroom 1	0.31	0.05	0.21	0.05	0.88	0.93	1.48
Classroom 2	0.24	0.03	0.13	0.07	0.55	0.84	0.45
Prepare room	0.54	0.07	0.30	0.12	1.07	0.50	−0.51
Storage	1.76	0.19	0.84	0.37	3.39	0.51	−0.54
Break	0.22	0.03	0.13	0.04	0.59	1.15	1.073
**2nd Sampling Campaign (March)**							
Classroom 1	0.31	0.04	0.16	0.06	0.72	0.93	1.37
Classroom 2	0.23	0.04	0.16	0.05	0.59	1.25	0.48
Prepare room	1.28	0.13	0.55	0.36	2.11	−0.07	−1.09
Storage	2.29	0.13	0.53	1.07	2.96	−1.29	1.26
Break	0.17	0.03	0.14	0.01	0.47	1.05	0.23
**3rd Sampling Campaign (May)**							
Classroom 1	0.47	0.10	0.46	0.01	1.39	0.92	−0.48
Classroom 2	0.30	0.04	0.18	0.01	0.62	0.09	−0.38
Prepare room	3.45	0.37	1.68	1.04	6.60	0.54	−0.95
Storage	2.90	0.24	1.09	1.20	5.31	0.64	−0.02
Break	0.03	0.01	0.02	0.01	0.08	1.12	−0.11

**Table 3:** Exposure to formaldehyde (CDI) and cancer risk (CR) of employees and students at the examined locations.

Employees							Students	
Sampling Site	Classroom 1	Classroom 2	Prepare Room	Storage Room	Break Room	Campaign Average	Classroom 1	Classroom 2
Exposed Group	Professors Laboratory Technicians	Professors Laboratory Technicians	Technical Assistants	Technical Assistants	All Employees	Cleaning Staff	Students	Students
1st Campaign								
C (ppm)	0.31	0.24	0.54	1.76	0.22	0.61	0.31	0.24
CA (mg∙m^−3^)	0.38	0.30	0.66	2.16	0.27	0.75	0,38	0,30
CDI (mg∙kg^−1^∙day^−1^) men	1.27 × 10^−2^	9.84 × 10^−3^	2.21 × 10^−2^	7.21 × 10^−2^	9.02 × 10^−3^	2.50 × 10^−2^	2.65 × 10^−5^	2.05 × 10^−5^
CR (40 years of exposure)	5.78 × 10^−4^	4.48 × 10^−4^	1.01 × 10^−3^	3.28 × 10^−3^	4.10 × 10^−4^	1.14 × 10^−3^	1.20 × 10^−6^	9.33 × 10^−7^
2nd Campaign								
C (ppm)	0.31	0.23	1.28	2.29	0.17	0.83	0.31	0.23
CA (mg∙m^−3^)	0.38	0.28	1.57	2.82	0.21	1.02	0.38	0.28
CDI (mg∙kg^−1^∙day^−1^) men	1.27 × 10^−2^	9.43 × 10^−3^	5.25 × 10^−2^	9.39 × 10^−2^	6.97 × 10^−3^	3.40 × 10^−2^	2.65 × 10^−5^	1.96 × 10^−5^
CR (40 years of exposure)	5.78 × 10^−4^	4.29 × 10^−4^	2.39 × 10^−3^	4.27 × 10^−3^	3.17 × 10^−4^	1.55 × 10^−3^	1.20 × 10^−6^	8.94 × 10^−7^
3rd Campaign								
C (ppm)	0.47	0.3	3.45	2.9	0.03	1.43	0.47	0.3
CA (mg∙m^−3^)	0.58	0.37	4.24	3.57	0.04	1.76	0.58	0.37
CDI (mg∙kg^−1^∙day^−1^) men	1.93 × 10^−2^	1.23 × 10^−2^	1.41 × 10^−1^	1.19 × 10^−1^	1.23 × 10^−3^	5.86 × 10^−2^	4.01 × 10^−5^	2.56 × 10^−5^
CR men (40 years of exposure)	8.77 × 10^−4^	5.60 × 10^−4^	6.43 × 10^−3^	5.41 × 10^−3^	5.60 × 10^−5^	2.67 × 10^−3^	1.83 × 10^−6^	1.17 × 10^−6^

## Data Availability

All data are presented in the paper (manuscript).
